# Supplementary material for: Identification and functional analysis of non-coding regulatory small RNA FenSr3 in Bacillus amyloliquefaciens LPB-18
Source: PeerJ. 2023 May 15;11:e15236. doi: 10.7717/peerj.15236 (PMC10194069; doi:10.7717/peerj.15236)
Supplement: Supplemental Information 4 [file peerj-11-15236-s004.zip › KO/CK-vs-T1_map/map05020.html]

KEGG PATHWAY: Prion diseases - Reference pathway


|  |  |
| --- | --- |
| **Prion diseases - Reference pathway** |  |

[
Pathway menu
| Organism menu
| Pathway entry
| Show description
| User data mapping
]

Prion diseases, also termed transmissible spongiform encephalopathies (TSEs), are a group of fatal neurodegenerative diseases that affect humans and a number of other animal species. The etiology of these diseases is thought to be associated with the conversion of a normal protein, PrPC, into an infectious, pathogenic form, PrPSc. The conversion is induced by prion infections (for example, variant Creutzfeldt-Jakob disease (vCJD), iatrogenic CJD, Kuru), mutations (familial CJD, Gerstmann-Straussler-Scheinker syndrome, fatal familial insomnia (FFI)) or unknown factors (sporadic CJD (sCJD)), and is thought to occur after PrPC has reached the plasma membrane or is re-internalized for degradation. The PrPSc form shows greater protease resistance than PrPC and accumulates in affected individuals, often in the form of extracellular plaques. Pathways that may lead to neuronal death comprise oxidative stress, regulated activation of complement, ubiquitin-proteasome and endosomal-lysosomal systems, synaptic alterations and dendritic atrophy, corticosteroid response, and endoplasmic reticulum stress. In addition, the conformational transition could lead to the lost of a beneficial activity of the natively folded protein, PrPC.

|  |  |  |
| --- | --- | --- |
| Reference pathway | 184% 150% 122% 100% 82% 67% 55% | 图片下载 |
